# Daily intake of up to two eggs for 11 weeks does not affect the cholesterol balance of Chinese young adults

**DOI:** 10.1002/fsn3.2734

**Published:** 2022-01-17

**Authors:** Zhili Ma, Wei Wu, Dexin Zhang, Ping Wu, Yuanhua Guo, Deyuan Li, Fang Yang

**Affiliations:** ^1^ 240515 School of Laboratory Medicine Hubei University of Chinese Medicine Wuhan China

**Keywords:** cardiovascular diseases risk, Chinese young adults, cholesterol balance, egg, satiety

## Abstract

Approximately 90% of the cholesterol content of the body is derived from de novo synthesis and the enterohepatic circulation. As numerous studies have shown previously, one egg per day intake has little impact of cholesterol balance in human body. Therefore, this study assumed that intake of up two eggs a day has little effect on biomarkers of cardiovascular diseases (CVDs) risk in Chinese young adults. With the increase in egg intake, total cholesterol, low‐density lipoprotein cholesterol (LDL‐C), high‐density lipoprotein cholesterol (HDL‐C), and choline all increased among all the groups as the study progressed from autumn to winter (*p* < .05). However, there were no differences in the plasma triglycerides, LDL‐C/HDL‐C ratio, glucose, liver enzymes, C‐reactive protein, and urinary microalbumin during the diet periods. Subjects who ate eggs at breakfast felt less hungry and more satisfied, which were relative with decreased fasting plasma ghrelin level (*p* < .05). Furthermore, egg‐derived cholesterol appeared to upregulate the mRNA levels of low‐density lipoprotein receptor and lecithin–cholesterol acyltransferase, and downregulate cholesteryl ester transfer protein and flavin‐containing monooxygenase 3 mRNA levels in isolated peripheral blood mononuclear cells. These results demonstrate that intake of up to two eggs a day had little effect on biomarkers of CVDs in young, healthy Chinese college students and provided useful evidence for the dietary guidelines regarding egg consumption.

## Graphical abstract

This study showed that a maximum of two eggs per day could not change the biomarkers associated with CVDs risk in young, healthy college students. Subjects who ate eggs at breakfast felt less hungry and more satisfied on the feeling of full day fullness.

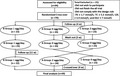

## 1 INTRODUCTION

The instability of cholesterol homeostasis in the body leads to hypercholesterolemia and further triggers the development of cardiovascular diseases (CVDs), which is a general term for many diseases, including heart disease, stroke, heart failure, arrhythmia, and heart valve problems. According to the China CVDs Report 2016, the current number of CVDs is 290 million, including 270 million hypertension, 13 million strokes, and 11 million coronary heart diseases (Bei et al., 2019). Epidemiological studies have shown that high concentrations of plasma cholesterol levels, especially low‐density lipoprotein cholesterol (LDL‐C), is directly positively correlated with CVDs, while high‐density lipoprotein cholesterol (HDL‐C) is negatively associated with CVDs (Chen & Levy, 2016). An increasing number of prospective cohort studies (Kurotani et al., 2014; Nakamura et al., 2006; Zazpe et al., 2011; Zhong et al., 2019), human intervention (DiMarco et al., 2017; Lemos et al., 2018; Missimer et al., 2017; Rueda & Khosla, 2013), and meta‐analytical evidence (Alexander et al., 2016; Godos et al., 2020; Rong et al., 2013; Shin et al., 2013; Tamez et al., 2016; Xu et al., 2019) ultimately lead to consensus: compared to other lifestyle factors, egg intake does affect cholesterol levels in the blood, but produce relatively small and clinically insignificant effects on the level of plasma LDL‐C/HDL‐C, the morbidity and mortality risk of stroke, heart disease, and CVDs in healthy people.

However, there seemed to be a positive relationship between egg intake and CVDs risk in patients with diabetic CVDs (Rong et al., 2013; Tamez et al., 2016; Zhong et al., 2019), uncertain relationship (Zazpe et al., 2011), or no relationship (Alexander et al., 2016; Kurotani et al., 2014). The reason for the inconsistency may be that volunteers in above studies came from different regions. Americans, predominantly on western diets, were more likely to have increased risk of CVDs, type 2 diabetes, or diabetes with CVD, than those who consume eggs in Asia, such as Chinese and Japanese. There were no significant or negative correlation between egg consumption and risk of CVDs, stroke, and type 2 diabetes in non‐U.S. regions, such as China, Japan, and Finland (Alexander et al., 2016; Kurotani et al., 2014; Tamez et al., 2016; Xu et al., 2019; Zazpe et al., 2011). Most of the above studies were from the western populations, only three were from Japan (Alexander et al., 2016; Nakamura et al., 2006; Shin et al., 2013) and two of the epidemiological survey and cohort study were from China (Qin et al., 2018; Xu et al., 2019), which worked on the effect of egg consumption on lipoprotein metabolism or the risk of CVDs. China is a big country in egg production and consumption. Since 1985, the total egg production of China has been ranked the first in the world for 35 consecutive years (Xu et al., 2020). Many Chinese have the habit of eating eggs every day. To confirm the assumption that intake of up to two eggs a day had little effect on biomarkers of CVDs in adults, we recruited 70 young, healthy Chinese college students to participate in a randomized crossover intervention.

## 2 MATERIALS AND METHODS

### 2.1 Materials

Hen eggs were purchased from Zhongbai Holdings Group Corporation (Wuhan, Hubei, China). Total plasma triglycerides (TG), total cholesterol (TC), HDL‐C, LDL‐C, glucose, alanine aminotransferase (ALT), aspartate aminotransferase (AST), C‐reactive protein (CRP), and urinary microalbumin kits were obtained from Daiichi Pure Chemicals. Plasma ghrelin kit was provided by Phoenix Pharmaceuticals. Plasma insulin kit was obtained from Mercodia AB and creatinine kit was obtained from Abcam. Total plasma apolipoprotein A‐I (apoA‐I) and apolipoprotein B (apoB) kits were obtained from Invitrogen. Ficoll‐Paque PREMIUM was obtained from GE Healthcare (1.077 g/ml). Ethylene diamine tetraacetic acid (EDTA) was obtained from Sigma‐Aldrich and TRIZOL reagent was obtained from Life Technologies corporation. The real‐time PCR Master Mix was provided by Toyobo and mRNA primers were obtained from Sango Biotech.

### 2.2 Subjects

Seventy healthy college students (34 males and 36 females) between 18 and 24 years of age were recruited to participate in a randomized crossover intervention study for 11 weeks. Participants were recruited from the Hubei University of Chinese Medicine with a body mass index (BMI) of 20.7 ± 2.3 kg/m^2^. Inclusion criteria included age (18–24 years), BMI (18.5–29.9 kg/m^2^), normal blood pressure (BP, 90/60–140/90 mmHg), healthy plasma lipid profiles, and willingness to consume the egg. Exclusion criteria included BMI ≥30 kg/m^2^, abnormal blood pressure, plasma TG >5.65 mmol/L, TC >6.21 mmol/L, and glucose >7.0 mmol/L. In addition, the exclusions also included current pregnancy or breastfeeding, egg allergy or celiac disease, hyperlipidemia, diabetes, fatty liver, obesity, and arteriosclerotic cardiovascular and cerebrovascular diseases caused by disorders of glucose and lipid metabolism.

### 2.3 Study design

Participants were randomly assigned to three groups, while group A did not eat eggs a day, group B had one egg a day, and group C had two eggs a day for 4 weeks. Following a 3‐week washout, participants in A and C groups crossed over to the alternate intervention for 4 weeks, while participants in B group consumed one egg per day until the end of the intervention. All egg intake interventions were carried out at breakfast and the eggs were standard medium‐ sized boiled hen eggs, which were cooked in our laboratory every morning. One large boiled egg contained about 0.5 g carbohydrate, 6.7 g protein, 4.4 g fat, 182 mg cholesterol, and 72 calories. Participants were divided into three groups and ate the boiled eggs at breakfast with noodles, rice, meat, bread, dairy products, vegetables, or fruits. The compliance of participant was administrated by staff members who went to the laboratory once a day to take the boiled eggs to their responsible group participants. During the intervention and washout periods, participants were asked to perform compliance issues strictly and avoid consuming whole eggs or foods containing predominately eggs after breakfast. All surveys occurred in the School of Laboratory Medicine, Hubei University of Chinese Medicine, Wuhan, China. The study was reviewed and approved by the Institutional Review Board at the Hubei University of Chinese Medicine (2018IEC(014)) and written informed consents were signed by participants before their participation in this study.

### 2.4 Dietary records, energy consumption, and satiety assessment

Two weeks before the intervention trial, all participants were not allowed to consume any food containing eggs. During this time, they have to finish the Dietary, Nutritional, and Health Questionnaire and the Food Frequency Questionnaire. Diet records were logged with 3‐day dietary intakes and physical activities by participants at baseline and each week during the intervention. Dietary intake includes all food, snacks, and beverages on two noncontinuous workdays and one weekend day. Fei‐hua Nutrition Calculator software v2.7.5.3 (Beijing Bowen News Technology Co., Ltd.) was used to analyze the dietary records, energy intake, and nutrients intake. The dietary cholesterol intake was calculated from the diet records. The energy consumption (EC) of participants was divided into three parts: basal metabolism (BM), physical activity (PA), and thermic effect of food (TEF). BM was calculated using body surface area, height, and body weight; PA was calculated using activity intensity and time; and TEF was 10% of BM. Satiety visual analog scales (VAS) and plasma ghrelin were used for analysis of satiety. The VAS questionnaire was examined before dinner following the method of Ratliff et al. (2010) with some modification. The plasma ghrelin was analyzed by ELISA kit.

### 2.5 Anthropometrics measures and blood and urine samples collection

Anthropometrics data of weight, BMI, waist circumference (WC), and BP were collected from participants at baseline and the end of each dietary period. An electronic scale was used to measure weight and height to the nearest 0.1 kg and 0.5 cm, respectively. BMI was calculated by dividing weight in kg by the square of height in meters (kg/m^2^). WC was measured on bare skin, at the top of the iliac crest to the nearest 0.1 cm. BP was measured with a portable automated blood pressure monitor BP‐223‐WH (Tanita Health Equipment Co.). After 12 h of fasting overnight, about 50 ml of blood was drawn at baseline, week 4, and week 11 in the morning time. Among them, 30 ml of blood was used to detect biochemical parameters and 20 ml for RT‐qPCR; 10 ml of urine was collected and placed at −80℃ for storage.

### 2.6 Plasma lipids, apolipoproteins, glucose, enzymes, ghrelin, creatinine, choline, and TMAO

The plasma TC, TG, HDL‐C, LDL‐C, glucose, ALT and AST levels, CRP, and urinary microalbumin were determined spectrophotometrically with AU5800 Series Clinical Chemistry Analyzers (Beckman Coulter, Inc.) using corresponding kits. Plasma apoA‐I and apoB were determined with colorimetric method by commercial kits. Plasma creatinine was also determined with an ELISA kit with colorimetric method. Plasma insulin was determined with an ELISA kit with cobas e 601 module (Roche Diagnostics). LDL‐C/HDL‐C and AST/ALT ratios were also calculated. Homeostatic model assessment for insulin (HOMA‐IR) was Fasting Glucose × Fasting Insulin/22.5, which was used to calculate insulin resistance (Lorenzo et al., 2012). According to the Modification of Diet in Renal Disease formula, estimated glomerular filtration rate (eGFR) was estimated (Levey et al., 2006). The plasma choline and trimethyl amine N‐oxide (TMAO) were assessed by ultraperformance liquid chromatography tandem mass spectrometry, with modifications based on instrumentation (DiMarco et al., 2017). Briefly, 30 μl of plasma samples was mixed with three volumes of acetonitrile containing 10 μmol/L d9‐choline chloride and d9‐TMAO (internal standards) for deproteinization, the supernatant was collected after centrifugation at 12,000 *g* for 10 min. Then, the samples (2 μl) were delivered with Agilent HPLC 1200 system equipped with SiO_2_ column (2.1 mm × 100 mm, 5 μm) and gradient eluted with 25% ammonium formate and 75% acetonitrile buffer (15 mmol/L, pH = 3.5) for separation. After electrospray ionization, the plasma choline and TMAO were determined by Agilent 6410A Triple Quad tandem mass spectroscopy equipped with multiple reaction monitoring mode.

### 2.7 Peripheral blood mononuclear cell isolation and quantitative RT‐qPCR

The peripheral blood mononuclear cells (PBMCs) were separated from the remaining 20 ml of fasting blood by density gradient centrifugation, using glycerol gradient Ficoll‐Paque PREMIUM based on the manufacturer's instructions. According to the manufacturer's protocol, total RNA was isolated from fresh PBMC by using TRIZOL reagent and estimated the concentration with a UV spectrophotometer at 260 and 280 nm. Total RNA was transcribed into cDNA and subjected to RT‐qPCR amplification with the One‐Step RT‐qPCR Quick Master Mix. Primer sequences were designed, which included flavin‐containing monooxygenase 3 (FMO3), cholesteryl ester transfer protein (CETP), lecithin–cholesterol acyltransferase (LCAT), paraoxonase 1 (PON1), and low‐density lipoprotein receptor (LDLR), shown in Table 1. The PCR went through 40 amplification cycles, and the annealing temperature used was 60–62°C. The expression of messenger RNA (mRNA) genes was measured by real‐ time PCR Master Mix (SYBR Green) with the Bio‐Rad C1000 Thermal Cycler (Bio‐Rad) and calculated with the $2^{‐\Delta \Delta C_{T}}$ threshold cycle method following normalization to β‐actin mRNA expression (Livak & Schmittgen, 2001).

**TABLE 1:** Primer sequences used for the RT‐qPCR analysis

Gene name	Accession number	Primer sequences (5′–3′)
β‐actin	NM_001101.5	F: GTCCACCGCAAATGCTTCTA
		R: TGCTGTCACCTTCACCGTTC
FMO3	NM_001002294.2	F: AGACACGAGTGGTCGGGAGA
		R: GCTGAATAGAAAAGCAGGTGGT
CETP	NM_000078.2	F: GCCTGCCCTCCTGGTGTT
		R: GCGATGGACAAGTGGCTGAT
LCAT	NM_000229.1	F: GCACTTTGAGGAAGGCTGGTA
		R: GCCGTGGTCGTAGATGTAGGT
PON1	NM_000446.6	F: ATTTCACCCGATGGCAAGTAT
		R: CAACCCAAAGGTCTCCTGTCTC
LDLR	NM_001195798.1	F: CTGGACCGTCGCCTTGCT
		R: CCATCGCAGACCCACTTGTA

Abbreviations: CETP, cholesteryl ester transfer protein; FMO3, flavin‐containing monooxygenase 3; LCAT, lecithin–cholesterol acyltransferase; LDLR, low‐density lipoprotein receptor; PON1, paraoxonase 1.

### 2.8 Statistical analyses

All the statistical analyses for the data were performed by using the software SPSS 24.0 (IBM SPSS Inc.). The statistical significance between the groups was assessed by paired *t* tests. All experiments were performed in triplicate and values are expressed as mean ± standard deviation. Probability values <.05 were considered statistically significant.

## 3 RESULTS

### 3.1 Study flow chart

**FIGURE 1:**
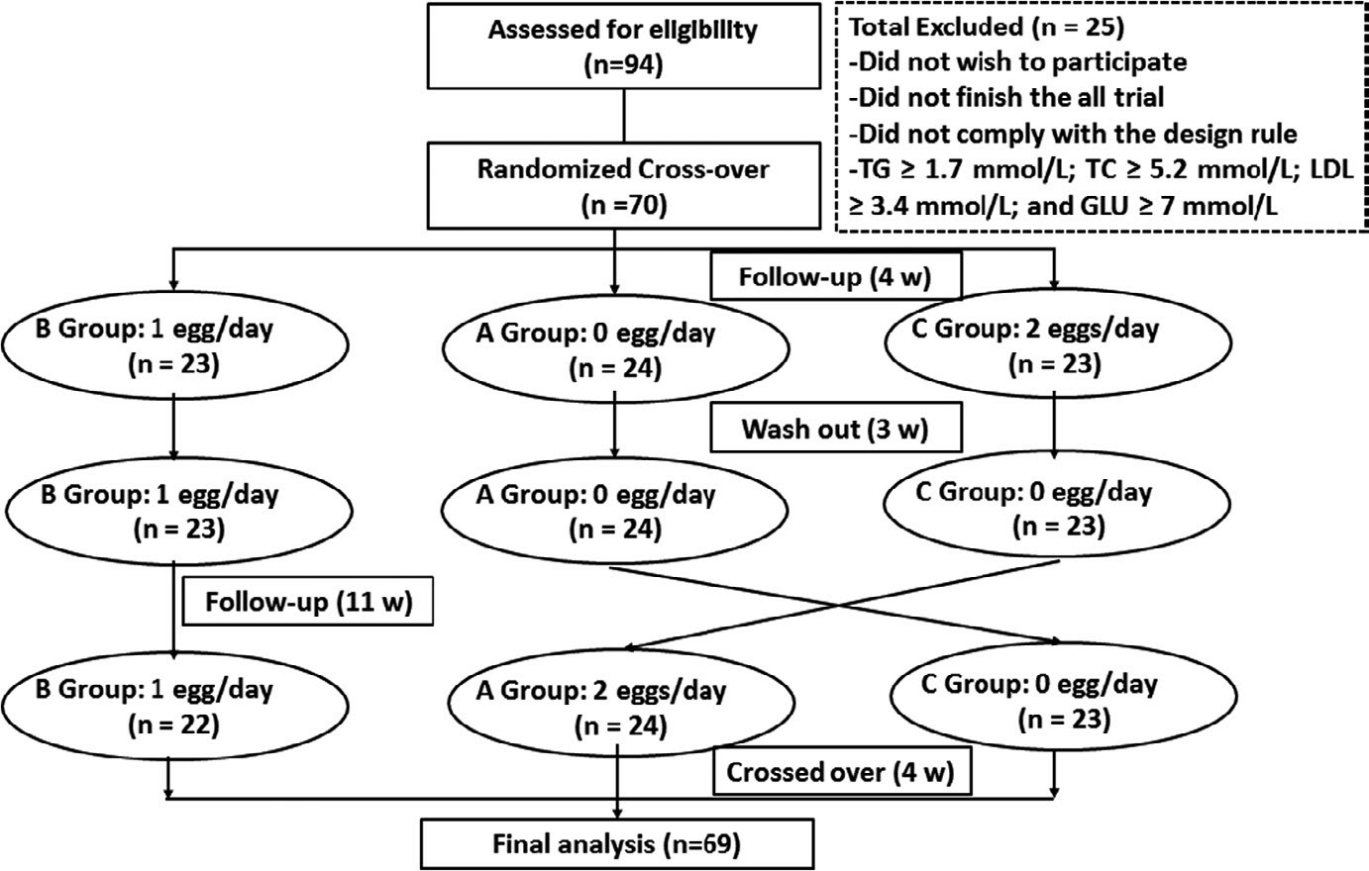
Flowchart of the study design. Sixty‐nine of 70 participants completed the study, one participant in group B dropped out of the study due to failed compliance while consuming one egg

At the beginning of the recruitment, 94 volunteers were willing to participate. Two weeks before the start of the formal trial, only 70 volunteers met the requirements and were willing to strictly comply with the intervention requirements after screening based on inclusion and exclusion criteria (Figure 1). There were 69 volunteers who completed the trial at the end of intervention, while only one participant in group B dropped out of the intervention during the third period due to fractured ankle.

### 3.2 Baseline characteristics

The baseline characteristics of 70 participants were shown in Table 2. The average age of 36 females and 34 males were 21.9 ± 0.8 years, and the BMI were 20.4 ± 2.3 kg/m^2^ and 20.9 ± 2.7 kg/m^2^, respectively. Participants had a healthy anthropometrics, with an average WC of 68.6 cm, body fat rate of 21.5%, and BP of 113/71 mmHg. The baseline plasma lipid profiles were within healthy ranges, with average TC <3.6 mmol/L, TG <0.8 mmol/L, LDL‐C < 2.2 mmol/L, HDL‐C > 1.3 mmol/L, and an average LDL‐C/HDL‐C of 1.4 ± 0.4. The average fasting glucose (GLU), fasting insulin (INS), and HOMA‐IR were 4.4 ± 0.3 mmol/L, 6.1 ± 2.6 mIU/L, and 1.2 ± 0.5, respectively. The baseline glucose profiles and CRP values indicated no significant glucose sensitivity or inflammation at baseline.

**TABLE 2:** Baseline characteristics of participants

Parameter	Female	Male	Total values
Gender	36	34	70
Age (years)	21.6 ± 0.5	22.2 ± 1.2	21.9 ± 0.8
BMI (kg/m^2^)	20.4 ± 2.3	20.9 ± 2.7	20.7 ± 2.3
Waist circumference (cm)	68.5 ± 5.2	77.8 ± 8.2	73.0 ± 8.2
Body fat rate (BFR, %)	25.9 ± 3.7	16.9 ± 3.8	21.5 ± 5.9
Systolic blood pressure (mmHg)	107.3 ± 13.2	118.8 ± 13.1	112.9 ± 14.3
Diastolic blood pressure (mmHg)	68.2 ± 9.2	73.9 ± 8.5	71.0 ± 9.3
Total cholesterol (TC, mmol/L)S	3.5 ± 0.6	3.4 ± 0.5	3.5 ± 0.5
LDL‐C (mmol/L)	1.9 ± 0.5	2.1 ± 0.4	2.0 ± 0.5
HDL‐C (mmol/L)	1.5 ± 0.3	1.3 ± 0.2	1.4 ± 0.3
LDL‐C/HDL‐C	1.3 ± 0.4	1.6 ± 0.4	1.4 ± 0.4
Triglycerides (TG, mmol/L)	0.7 ± 0.2	0.7 ± 0.2	0.7 ± 0.2
ApoA‐I (g/L)	1.1 ± 0.2	1.0 ± 0.1	1.1 ± 0.1
ApoB (g/L)	0.7 ± 0.2	0.7 ± 0.2	0.7 ± 0.2
Plasma ghrelin (pg/mL)	675 ± 124	691 ± 147	683 ± 136
Fasting glucose (GLU, mmol/L)	4.4 ± 0.2	4.5 ± 0.3	4.4 ± 0.3
Fasting insulin (INS, mIU/L)	5.9 ± 2.4	6.2 ± 2.7	6.1 ± 2.5
HOMA‐IR	1.2 ± 0.5	1.3 ± 0.6	1.2 ± 0.5
C‐reactive protein (CRP) (mg/L)	0.5 ± 0.2	1.0 ± 0.3	0.7 ± 0.2
Creatinine (μmol/L)	57.8 ± 7.0	71.7 ± 6.4	64.6 ± 6.8
eGFR (ml/min)	114.4 ± 21.2	130.6 ± 22.0	122.2 ± 22.9
Alanine aminotransferase (ALT, U/L)	8.3 ± 2.6	15.4 ± 8.7	11.8 ± 7.3
Aspartate aminotransferase (AST, U/L)	12.7 ± 2.6	14.8 ± 4.6	13.7 ± 3.8
AST/ALT	1.6 ± 0.4	1.1 ± 0.5	1.4 ± 0.5
Choline (nmol/mL)	5.5 ± 0.8	5.7 ± 1.1	5.6 ± 1.0
Trimethyl amine *N*‐oxide (TMAO) (μmol/L)	2.8 ± 1.4	3.0 ± 1.9	2.9 ± 1.7
Urinary microalbumin (mALB, mg/L)	30.5 ± 2.4	8.6 ± 1.1	19.9 ± 1.8

Abbreviations: ApoA‐I, plasma apolipoprotein A‐I; ApoB, apolipoprotein B; BMI, body mass index; eGFR, estimated glomerular filtration rate; HDL‐C, high‐density lipoprotein cholesterol; HOMA‐IR, homeostatic model assessment for insulin resistance; LDL‐C, low‐density lipoprotein cholesterol.

### 3.3 Dietary records after a 4‐week intervention

Dietary records, EC, protein, carbohydrate, fat, cholesterol, lutein, zeaxanthin, minerals, and vitamins were recorded and calculated as illustrated in Table 3. While there was no significant difference in weight after the 4‐week intervention, subjects that consumed one or two eggs had higher EC and lower energy intake (*p* < .01). Subjects in B (1 egg/day) and C (2 eggs/day) groups consumed a greater amount of protein, fat (cholesterol), lutein, zeaxanthin, vitamins A and E (*p* < .01), and a less amount of carbohydrate and vitamin C (*p* < .01) than subjects in A (0 egg/day) group and baseline.

**TABLE 3:** Dietary records after a 4‐week intervention

Parameter	Baseline	A (0 egg/day)	B (1 egg/day)	C (2 eggs/day)
Weight (kg)	58.6 ± 9.5	59.6 ± 10.9	58.8 ± 9.4	59.7 ± 8.5
Energy consumption (EC, kcal)	2078 ± 434^b^	2067 ± 371^b^	2171 ± 656^a^	2145 ± 830^a^
Energy intake (EI, kcal)	1823 ± 628^ab^	1881 ± 689^a^	1848 ± 637^a^	1776 ± 849^b^
Energy balance (kcal)	−255 ± 53^ab^	−186 ± 48^a^	−323 ± 64^bc^	−369 ± 67^c^
Protein (g/day)	63.0 ± 27.2^bc^	61.6 ± 28^c^	68.0 ± 28.2^ab^	72.3 ± 25.3^a^
Carbohydrate (g/day)	283.0 ± 60.7^a^	297.7 ± 88.6^a^	273.9 ± 95.2^ab^	249.3 ± 78.9^b^
Total fat (g/day)	48.8 ± 32.8^b^	49.3 ± 27.9^b^	53.4 ± 27.9^a^	54.4 ± 34.3^a^
Cholesterol (mg/day)	156.4 ± 75.3^c^	162.1 ± 81.7^c^	346.5 ± 74.9^b^	496.8 ± 146.3^a^
Lutein + zeaxanthin (µg/day)	1965 ± 2321^b^	2231 ± 1864^b^	2663 ± 3185^ab^	3016 ± 3375^a^
Ca (mg/day)	503.1 ± 179.8	478.6 ± 225.2	512.7 ± 214.3	485.4 ± 183.9
Fe (mg/day)	20.7 ± 8.6	20.6 ± 7.2	22.5 ± 9.1	19.8 ± 8.3
Zn (mg/day)	11.9 ± 4.7	10.1 ± 4.4	11.8 ± 5.2	12.5 ± 5.9
V_A_ (µg/day)	390.2 ± 223.1^b^	385.6 ± 176.0^b^	509.2 ± 248.3^a^	594.7 ± 274.6^a^
V_B1_ (mg/day)	0.9 ± 0.5	0.9 ± 0.6	0.8 ± 0.6	0.9 ± 0.5
V_B2_ (mg/day)	0.8 ± 0.6	0.9 ± 0.6	0.9 ± 0.5	0.9 ± 0.6
V_C_ (mg/day)	52.6 ± 33.1^ab^	55.5 ± 40.9^a^	51.9 ± 37.5^b^	49.0 ± 40.1^b^
V_D_ (mg/day)	3.5 ± 2.4	3.6 ± 2.2	3.7 ± 3.4	3.7 ± 3.2
V_E_ (α‐tocopherol, mg/day)	12.7 ± 3.8^c^	13.2 ± 5.2^b^	14.3 ± 4.8^ab^	15.4 ± 3.5^a^

Note

Two weeks before the intervention trial, participants were not allowed to consume any food containing eggs. During this time, they have to finish the Dietary, Nutritional, and Health Questionnaire and the Food Frequency Questionnaire. Logs were provided to subjects to record 3‐day dietary intakes and physical activity at baseline and the last day of the first intervention (4 weeks). The energy consumption (EC) of participants was calculated by basal metabolism, physical activity, and thermic effect of food. Fei‐hua Nutrition Calculator software v2.7.5.3 was used to analyze the dietary records, energy intake, macronutrient, micronutrient, cholesterol, and carotenoid intake. Each value is presented as mean ± *SD* of three independent experiments (a > b > c, all *p*s < .05); *n* = 70.

### 3.4 Effects of eggs intervention on satiety

**FIGURE 2:**
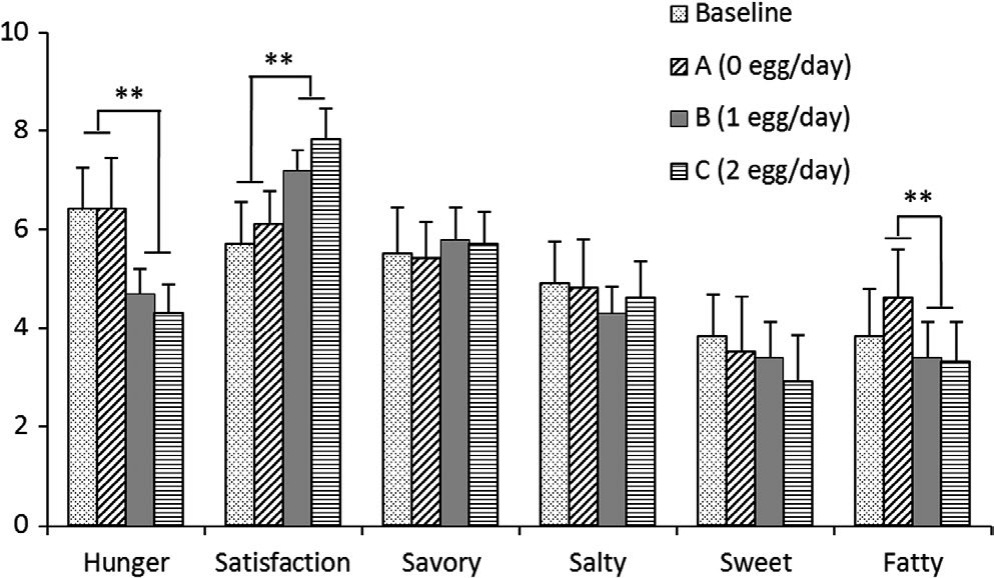
Satiety VAS for participants after a 4‐week egg intervention. Two weeks before the intervention trial, participants were not allowed to consume any food containing eggs. Satiety was analyzed by subjective (satiety visual analog scales, VAS) at baseline and each week during the first intervention (4 weeks). Satiety scales were completed prior to dinner. **p < .01 were compared to lower values. Each value is presented as mean ± *SD* of three independent experiments; *n* = 70

The VAS analysis showed that subjects who ate eggs for breakfast felt less hungry and more satisfied before eating dinner than subjects who did not eat eggs (Figure 2, *p* < .01). Accordingly, fasting plasma ghrelin, an objective measure of satiety, were significantly decreased as compared to breakfast without egg and baseline in Table 4 (*p* < .01). It showed that the delayed effect of consuming eggs at breakfast on the feeling of full day fullness. Finally, subjects consuming eggs felt less of a desire for something fatty prior to dinner (*p* < .01).

**TABLE 4:** Anthropometrics, blood pressure, and plasma lipids after a 11‐week egg intervention

Parameter	A (0W)	B (0W)	C (0W)	A (4W)	B (4W)	C (4W)	A (11W)	B (11W)	C (11W)
Gender (F : M)	12:12	15:8	9:14	12:12	15:8	9:14	12:12	14:8	9:14
BMI (kg/m^2^)	20.1 ± 2.3^b^	20.7 ± 2.3^ab^	21.3 ± 2.2^a^	20.4 ± 2.2^b^	20.9 ± 2.3^ab^	21.6 ± 2.2^a^	20.8 ± 2.5^ab^	20.8 ± 2.4^ab^	21.5 ± 2.4^a^
Waist circumference (cm)	73.8 ± 7.8	71.4 ± 7.8	73.8 ± 6.9	73.6 ± 7.5	71.6 ± 7.5	76.4 ± 7.0	74.2 ± 9.2	72.0 ± 7.1	76.8 ± 7.8
Systolic blood pressure (mmHg)	112.9 ± 11.3	110.8 ± 16.0	115.0 ± 15.5	109.5 ± 9.3	108.2 ± 10.3	110.7 ± 8.6	110.0 ± 9.8	114.2 ± 12.7	114.7 ± 15.7
Diastolic blood pressure (mmHg)	71.2 ± 8.3	69.5 ± 10.2	72.2 ± 9.5	69.7 ± 6.1	66.6 ± 7.9	72.2 ± 7.8	75.5 ± 7.9	72.6 ± 8.0	78.4 ± 9.4
Total cholesterol (TC, mmol/L)	3.4 ± 0.4^b^	3.6 ± 0.5^ab^	3.4 ± 0.6^b^	3.6 ± 0.5^ab^	3.8 ± 0.5^a^	3.8 ± 0.5^a^	4.0 ± 0.6^a^	3.9 ± 0.5^a^	3.6 ± 0.6^ab^
LDL‐C (mmol/L)	1.9 ± 0.4	2.0 ± 0.4	2.0 ± 0.6	2.0 ± 0.5	2.1 ± 0.4	2.1 ± 0.5	2.3 ± 0.5	2.2 ± 0.6	2.1 ± 0.6
HDL‐C (mmol/L)	1.5 ± 0.3^b^	1.5 ± 0.3^b^	1.3 ± 0.3^c^	1.5 ± 0.3^b^	1.6 ± 0.4^a^	1.6 ± 0.2^ab^	1.7 ± 0.3^a^	1.7 ± 0.4^a^	1.5 ± 0.2^b^
LDL‐C/HDL‐C	1.4 ± 0.4	1.4 ± 0.4	1.6 ± 0.5	1.4 ± 0.5	1.4 ± 0.4	1.3 ± 0.5	1.4 ± 0.4	1.3 ± 0.5	1.4 ± 0.5
Triglycerides (TG, mmol/L)	0.7 ± 0.2	0.7 ± 0.2	0.7 ± 0.2	0.7 ± 0.2	0.8 ± 0.3	0.8 ± 0.2	0.9 ± 0.3	0.8 ± 0.2	0.8 ± 0.2
ApoA‐I (g/L)	1.1 ± 0.1^b^	1.1 ± 0.1^b^	1.1 ± 0.1^b^	1.3 ± 0.2^ab^	1.4 ± 0.2^ab^	1.5 ± 0.1^a^	1.5 ± 0.2^a^	1.6 ± 0.3^a^	1.4 ± 0.1^ab^
ApoB (g/L)	0.7 ± 0.1	0.7 ± 0.1	0.7 ± 0.1	0.7 ± 0.2	0.8 ± 0.1	0.7 ± 0.2	0.9 ± 0.1	0.8 ± 0.2	0.9 ± 0.1
Plasma ghrelin (pg/mL)	682 ± 115^a^	688 ± 128^a^	681 ± 119^a^	697 ± 127^a^	598 ± 163^b^	504 ± 134^c^	513 ± 142^c^	591 ± 125^b^	695 ± 147^a^

Note

After 2 weeks of not consuming any food containing eggs, all participants were gathered to draw blood and collect urine after 12‐hr overnight fast at baseline, week 4, and week 11 in the morning time. The plasma triglycerides, total cholesterol, HDL‐C, and LDL‐C were determined spectrophotometrically using appropriate kits (Daiichi Pure Chemicals Co.). Total ghrelin was quantified using a sandwich ELISA kit and plasma apoA‐I and apoB were determined with a commercially available multiplex kit (Invitrogen). Anthropometrics, blood pressure, and waist circumference were noted from participants at the same day. BMI was calculated by dividing weight in kg by the square of height in meters (kg/m^2^). WC was measured on bare skin, at the top of the iliac crest to the nearest 0.1 cm. Blood pressure was measured with a portable automated blood pressure monitor BP‐223‐WH (Tanita Health Equipment Co.). Each value is presented as mean ± *SD* of three independent experiments (a > b > c, all *p* < .05); *n* = 69. Abbreviations: ApoA‐I, plasma apolipoprotein A‐I; ApoB, apolipoprotein B; BMI, body mass index; LDL‐C, low‐density lipoprotein cholesterol; HDL‐C, high‐density lipoprotein cholesterol.

### 3.5 Effects of eggs intervention on anthropometrics, blood pressure, and plasma lipids

There were no differences in anthropometric data such as BMI, WC, and BP among the intervention. All participants maintained normal BP levels and systolic BP in three groups kept under 120 mmHg, as shown in Table 4. BMI slightly increased in all groups as the study progressed from autumn to winter, probably because all participants increased their diet with decreased exercise in cold weather (Table 4). But there is no significant difference in body weight and BMI changes among the three groups during the same intervention period, which also indicates that the egg consumption does not raise the risk of obesity. This distribution could also be seen from the blood lipid levels of the participants, TC, HDL‐C, and apoA‐I all increased with the changed time among all the groups (*p* < .01), while the TG, LDL‐C, LDL‐C/HDL‐C, and apoB did not produce any significant effect.

### 3.6 Effects of eggs intervention on fasting glucose, C‐reactive protein, creatinine, liver enzymes, and urinary microalbumin

Several studies suggest with a cut‐off of HOMA‐IR >2 for any insulin resistance, there were no significant effects of the egg intervention on plasma glucose profiles in Table 5. In contrast, plasma insulin and HOMA‐IR significantly increased from baseline to week 11 in all participants (*p* < .01). When analyzed separately in 0, 4, and 11 weeks, participants in three groups had no significant increases in plasma insulin and HOMA‐IR over time. In the same situation, creatinine in plasma significantly increased with time in all participants (*p* < .01), but no significant changes at the same period among three groups, while significant changes were not observed in CRP, eGFR, ALT, AST, AST/ALT, and urinary microalbumin (Table 5).

**TABLE 5:** Fasting glucose, creatinine, liver enzymes, and urinary microalbumin after a 11‐week egg intervention

**Parameter**	**A (0W)**	**B (0W)**	**C (0W)**	**A (4W)**	**B (4W)**	**C (4W)**	**A (11W)**	**B (11W)**	**C (11W)**
Fasting glucose (GLU, mmol/L)	4.3 ± 0.2	4.4 ± 0.2	4.5 ± 0.4	4.3 ± 0.4	4.2 ± 0.5	4.4 ± 0.3	4.2 ± 0.5	4.3 ± 0.3	4.3 ± 0.3
Fasting insulin (INS, mIU/L)	5.6 ± 2.4^c^	6.2 ± 2.4^bc^	6.4 ± 2.7^bc^	7.7 ± 1.9^ab^	7.5 ± 3.9^ab^	7.1 ± 2.3^b^	7.5 ± 2.5^ab^	7.4 ± 3.4^ab^	8.6 ± 4.8^a^
HOMA‐IR	1.1 ± 0.5^c^	1.2 ± 0.5^bc^	1.3 ± 0.6^b^	1.5 ± 0.4^ab^	1.4 ± 0.8^ab^	1.4 ± 0.5^ab^	1.4 ± 0.5^ab^	1.4 ± 0.6^ab^	1.6 ± 0.9^a^
C‐reactive protein (CRP) (mg/L)	0.9 ± 0.3	0.5 ± 0.2	0.8 ± 0.2	0.8 ± 0.4	0.7 ± 0.2	1.0 ± 0.3	0.9 ± 0.3	0.5 ± 0.2	0.5 ± 0.3
Creatinine (μmol/L)	64.6 ± 9.9^a^	63.6 ± 10.5^a^	65.6 ± 8.9^a^	69.3 ± 9.9^ab^	66.1 ± 7.1^a^	70.8 ± 9.9^ab^	72.1 ± 10.3^b^	66.9 ± 15.0^ab^	74.0 ± 10.9^b^
eGFR (ml/min)	122.2 ± 23.7	121.4 ± 23.4	123.3 ± 22.5	115.6 ± 21.1	117.1 ± 18.4	116 ± 21.0	113.4 ± 20.1	132.0 ± 23.9	101.2 ± 16.3
Alanine aminotransferase (ALT, U/L)	10.1 ± 5.2	10.1 ± 4.4	14.0 ± 9.9	11.4 ± 6.1	11.1 ± 4.5	14.6 ± 8.7	14 ± 7.0	12.6 ± 7.1	19.9 ± 8.9
Aspartate aminotransferase (AST, U/L)	13.1 ± 2.3	13.3 ± 3.7	14.8 ± 5.0	13.7 ± 2.6	13.7 ± 2.8	14.6 ± 3.9	15 ± 4.2	14.7 ± 3.3	17.5 ± 3.5
AST/ALT	1.5 ± 0.6	1.4 ± 0.4	1.2 ± 0.4	1.4 ± 0.5	1.4 ± 0.4	1.2 ± 0.4	1.2 ± 0.4	1.4 ± 0.6	1.2 ± 0.5
Urinary microalbumin (mALB, mg/L)	14.2 ± 6.4	12.9 ± 4.8	12.9 ± 6.4	18.8 ± 2.7	15.8 ± 1.7	14.9 ± 5.3	8.9 ± 2.4	10.4 ± 4.0	13.0 ± 2.1

Note

After 2 weeks of not consuming any food containing eggs, all participants were gathered to draw blood and collect urine samples after a 12‐hr overnight fast at baseline, week 4, and week 11 in the morning time. The plasma glucose, alanine aminotransferase (ALT) and aspartate aminotransferase (AST) levels, C‐reactive protein (CRP), and urinary microalbumin were determined spectrophotometrically using appropriate kits (Daiichi Pure Chemicals Co., Tokyo, Japan). Plasma creatinine was determined with an ELISA kit from Abcam, MA and insulin was determined with an ELISA kit from Mercodia AB, Uppsala, Sweden. Homeostatic model assessment for insulin resistance (HOMA‐IR) was Fasting Glucose ×Fasting Insulin / 22.5. Estimated glomerular filtration rate (eGFR) was calculated using the Modification of Diet in Renal Disease (MDRD) formula. Each value is presented as mean ± *SD* of three independent experiments (a > b > c, all *p* <.05); *n* = 69. Abbreviations: HOMA‐IR, homeostatic model assessment for insulin resistance; eGFR, estimated glomerular filtration rate.

### 3.7 Effects of eggs intervention on plasma choline and TMAO

**FIGURE 3:**
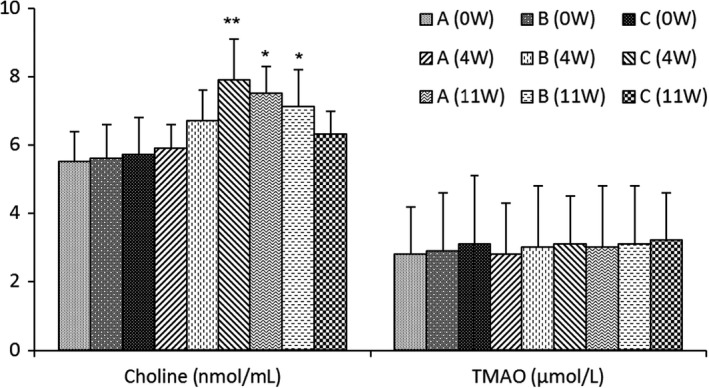
Concentrations of plasma choline and trimethyl amine N‐oxide (TMAO) for participants after a 11‐week egg intervention. After 2 weeks of not consuming any food containing eggs, all participants were gathered to draw blood and collect urine samples after a 12‐hr overnight fast at baseline, week 4, and week 11 in the morning time. The plasma choline and TMAO were determined by ultraperformance liquid chromatography tandem mass spectrometry (UPLC‐MS/MS). *p < .05, **p < .01 were compared to lower values. Each value is presented as mean ± *SD* of three independent experiments; *n* = 69

After consuming two eggs a day, there were significant increases in plasma choline in group C (4W) and group A (11W), also in group B (11 W) following the consumption of one egg per day during the whole trial (*p* < .01) (Figure 3). However, there was no difference in plasma TMAO levels in all groups for the duration of the intervention.

### 3.8 Effects of eggs intervention on PBMCs gene expression

**FIGURE 4:**
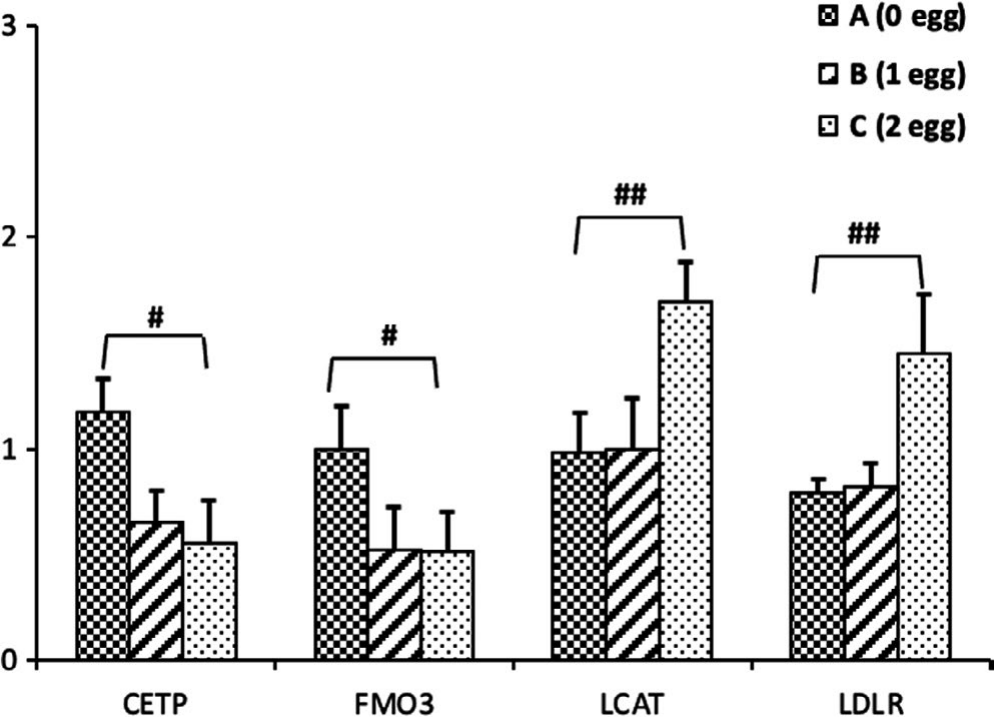
The effects of eggs intervention on mRNA expression levels of cholesteryl ester transfer protein (CETP), flavin‐containing monooxygenase 3 (FMO3), lecithin–cholesterol acyltransferase (LCAT), and low‐density lipoprotein receptor (LDLR) for participants after a 4‐week egg intervention. Values of the indicated genes were normalized to that of β‐actin as a housekeeping gene using the $2^{‐\Delta \Delta C_{T}}$ threshold cycle method, which was set to 1. ^#^ *p* < .05, ^##^ *p* < .01 were compared to control group A (0 egg). Each value is presented as mean ± *SD* of three independent experiments; *n* = 24. Abbreviations: CETP, cholesteryl ester transfer protein; FMO3, flavin‐containing monooxygenase 3; LCAT, lecithin–cholesterol acyltransferase; LDLR, low‐density lipoprotein receptor

To determine whether egg intake would affect the expression of genes related to lipoprotein metabolism and TMAO formation, we measured PBMCs gene expression of the LDLR, CETP, LCAT, PON1, and FMO3 (Figure 4). The expression of CETP and FMO3 were lower with eggs intervention than non‐egg intervention (group A) (*p* < .01), however, the expression of LCAT and LDLR in group C with two eggs per day were significantly higher than groups A (0 egg per day) and B (one egg per day) (*p* < .01). Furthermore, the relative mRNA expression of PON1 among the three groups was very low and no differences were seen in all participants.

## 4 DISCUSSION

Since eggs are the main source of dietary cholesterol, nutritionists in the past recommended that healthy people eat up to two to four eggs per week in prevention of CVDs (McNamara, 2015). At present, there are not too many restrictions on the egg intake recommendations for healthy adults except that of the Mayo Clinic suggestion of consuming no more than 300 mg of cholesterol a day (Lopez‐Jimenez, 2014). For patients diagnosed with CVDs, only the 2016 Chinese Guideline for the Management of Dyslipidemia in adults, the Mayo Clinic, and the German Heart Association (Deutsche Herzstiftung) recommended limiting the intake of eggs and dietary cholesterol (Xu et al., 2020). The American Heart Association claimed that no upper limit for either egg or dietary cholesterol in 2013 (American College of Cardiology & American Heart Association, 2014) and the Dietary Guidelines for Americans 2015 also removed the 300 mg/day limit of dietary cholesterol 2 years later (U.S. Department of Health and Human Services & U.S. Department of Agriculture, 2014). The Dietary Guidelines for Chinese 2016 mentioned that eggs have high nutritional value. It is recommended to take one egg per day (equivalent to about 50 g), and do not discard the yolk when eating eggs (Wang et al., 2016).

According to the Dietary Guidelines for Chinese 2016, we evaluated that the effects of consuming up to two eggs per day on lipid metabolism‐related biomarkers of CVDs risk in young, healthy college students in China. In this study, we demonstrated that up to two eggs per day did not produce significant effects on blood pressure, TG, LDL‐C/HDL‐C, apoB, and fasting glucose during the two interventional periods (0–4 weeks and 8–11 weeks), while TC, HDL‐C, and apoA‐I all increased with the changed time among all the groups as the study progressed from autumn to winter (*p* < .05), probably because all participants increased their diet with decreased exercise in cold weather. In addition to diet and exercise, slight weight gain without significant difference in groups A and C might be related to sleep, mood, hormones, and alcohol, which are also important factors that were not considered in this study. Besides, subjects consuming eggs felt less desire for something fatty prior to dinner and the delayed effect of consuming eggs at breakfast on the feeling of full day fullness.

Eggs are low‐cost, but nutrient‐rich food that provides balanced nutrients that affect human health. It has been reported that the diet rich in saturated fatty acids intake would induce raised plasma TC and LDL‐C levels (Rivellese et al., 2003); however, elevation of unsaturated fatty acids intake lowered LDL‐C without affecting HDL‐C (Gill et al., 2003; Rivellese et al., 2003), or increased the HDL‐C level (Mensink & Katan, 1992), or led to a decrease in TC and LDL‐C levels (Haban et al., 2004). We demonstrated that up to two eggs per day did increase plasma TC, HDL‐C, and LDL‐C levels during the interventional periods in a young, healthy college student. However, raised HDL‐C resulted in the maintenance of the LDL‐C/HDL‐C ratio, which is a key indicator of CVDs. So that, not only absolute concentrations of total lipids, but also the balance of fatty acids, in particular, are crucial to our health. A prospective cohort study has reported that replacing 5% of energy intake from saturated fatty acids with monounsaturated fatty acids has shown a 15% reduction in CVDs risk (Li et al., 2015). Besides, in subjects who took one or two eggs had more plasma HDL‐C and apoA‐I levels than subject who did not eat eggs, which demonstrated that the high content of phosphatidylcholine in eggs participates in the assembly of HDL and the efflux of cholesterol after ingestion.

Qin et al. (2018) investigated over 0.5 million Chinese adults aged 30–79 years, showed that egg intake (<1 egg per day) was associated with a lower risk of CVD, IHD, MCE, hemorrhagic stroke, and ischemic stroke in middle‐aged Chinese adults. Xu et al. (2019) recruited 28,024 health volunteers without CVDs from Guangzhou Biobank cohort study and showed that there were no significant differences in CVDs or all‐cause mortality between over seven eggs per week intake and less than one egg per week intake after 9.8 years follow‐up. In addition, Japanese research did not support the association between egg intake and the CHD risk (Nakamura et al., 2004, 2006). On the contrary, increased egg intake tends to reduce the risk of stroke (Sauvaget et al., 2003). However, Zhong et al. (2019) reported that there is a significant correlation between higher consumption of dietary cholesterol or eggs and the risk of CVDs and all‐cause mortality in a dose–response manner among U.S. adults. The reason for the inconsistency may be that volunteers in above studies came from different regions. Compared with Asians (such as Chinese and Japanese), Americans who eat eggs on a Western diet may have slightly increased risk of CVDs, type 2 diabetes, or CVDs diabetes.

Accumulation of TMAO in the body could promote lipid accumulation, foam cell formation, and atherosclerosis, then increase the incidence of CVDs, and even death (Al‐Obaide et al., 2017; Koeth et al., 2013). This is because choline is converted into trimethylamine (TMA) by gut microbiota, and then transformed into TMAO by flavin monooxygenases (FMO), which is expressed in the liver. Even after adjusting for other known risks, the concentration of TMAO in plasma seems to be a powerful indicator for predicting the overall mortality of CVDs patients. Our research found that although there was a significant increase in the plasma choline level after egg consumption (*p* < .05), no difference was found in TMAO levels for the duration of the intervention among the three groups. This result is consistent with previous reports that there was no significant increase in fasting plasma TMAO concentration in healthy, young people after egg consumption (DiMarco et al., 2017; Tamez et al., 2016). Phospholipids, especially phosphatidylcholine and choline, are everywhere in our diet, and eliminating choline means reducing the intake of more healthy food. Egg phosphatidylcholine can be degraded into phosphatidic acid and choline by phospholipase D. Choline is important in the formation of the neurotransmitter, methyl group donor, acetylcholine, phospholipids, and betaine, making it an essential dietary component for humans at all stages of life (Wiedeman et al., 2018). Egg phosphatidylcholine and sphingomyelin seem to modulate cholesterol absorption and inflammation in preclinical studies, while the consumption of egg phospholipids has been linked to positive alterations in biomarkers related to HDL reverse cholesterol transport in clinical studies (Blesso, 2015; Yang et al., 2018). The effect of egg choline on human CVDs risk and TMAO concentration is very complicated, and further research is needed.

In addition, the exogenous cholesterol from eggs seems to upregulate the mRNA levels of LDLR and LCAT, and downregulate CETP and FMO3 mRNA levels in isolated PBMCs. LDLR is the main receptor acting in the cellular uptake of cholesterol in circulating lipoproteins. LCAT promotes the HDL pool maturation and the cholesterol outflow from peripheral tissues (Jonas, 2000). CETP is an enzyme responsible for moving cholesterol esters and TG from HDLs to LDLs that will cause the lipoproteins deposition disorder and arteriosclerosis development by reducing HDL levels (Tárraga et al., 2019). The high LCAT and low CETP mRNA expressions in subjects consuming eggs showed that the egg diet promoted HDL maturation and accelerated the removal of cholesterol from the circulation. This inference was consistent with the low LDL‐C and the high HDL‐C levels of plasma in subjects consuming eggs, compared with subjects consuming no egg. Therefore, reverse cholesterol transport is one of several proposed mechanisms for HDL to provide protection from CVDs (Hoeg et al., 1996). Missimer et al. (2017) also reported that dietary cholesterol from eggs can reduce the cholesterol biosynthesis in the liver, which leads to the regulation of extrahepatic cholesterol synthesis. FMO3 transforms TMA into TMAO, which promotes lipid accumulation and foam cell formation to atherosclerosis (Al‐Obaide et al., 2017). Although the mRNA expressions of FMO3 were lower in subjects consuming eggs, the level of TMAO in plasma is not affected by eating eggs. The metabolism of TMAO is more complicated, and it may also be affected by other components in the egg. This needs to be further studied in the future.

A good diet does not only focus on one nutrient or one food. Contemporary nutrition emphasizes the health of the overall diet, such as eating more fresh vegetables and fruits, whole grains, nuts, low‐fat dairy products, and eating 2–3 times fish or seafood, eat less red meat, eat less or no processed meat, fried food, control the intake of salt, oil, and sugar. It may be relatively more important to pay attention to the health of the overall eating pattern. The new Dietary Guidelines for Americans (2020–2025) put this emphasis on the importance of the overall healthy eating pattern, rather than individual nutrition or food alone (U.S. Department of Health and Human Services & U.S. Department of Agriculture, 2020). For Chinese people, especially children and adolescents in the growth and development period or people who are poor and struggling with food and clothing, eggs may be a perfect food as a source of healthy diet because they are cheap, with high‐quality protein, unsaturated fatty acids, and various important nutrients, such as vitamins, iron, zinc, and selenium.

## 5 CONCLUSION

Diet is an important modifiable factor that can affect the risk of CVDs and the progression of atherosclerosis. Eggs are rich in essential proteins, fats, vitamins, minerals, and biologically active ingredients, which can provide balanced nutrients that have an impact on human health. This study showed that a maximum of two eggs per day could not change the biomarkers associated with CVDs risk in young, healthy college students and provided useful evidence for the dietary guidelines regarding egg consumption.

## CONFLICT OF INTEREST

All authors declare that there are no conflicts of interest.

## Data Availability

The data used to support the findings of this study are available from the corresponding author upon request.
